# SERPINB5/TGF-**β** signaling modulates desmoplakin membrane localization and ameliorates pemphigus vulgaris skin blistering

**DOI:** 10.1172/jci.insight.183024

**Published:** 2025-10-02

**Authors:** Maitreyi Rathod, Mariam Petrosyan, Aude Zimmermann, Maike Märker, Tobias Gosau, Henriette Franz, Tomás Cunha, Dario Didona, Michael Hertl, Enno Schmidt, Volker Spindler

**Affiliations:** 1 Institute of Anatomy and Experimental Morphology, University Medical Centre-Eppendorf, Hamburg, Germany.; 2 Department of Biomedicine, University of Basel, Basel, Switzerland.; 3 Department of Dermatology and Allergology, Philipps-University Marburg, Marburg, Germany.; 4 Lübeck Institute of Experimental Dermatology, University of Lübeck, Lübeck, Germany.; 5 Department of Dermatology, University Hospital of Schleswig-Holstein, Campus Lübeck, Lübeck, Germany.

**Keywords:** Cell biology, Dermatology, Cell migration/adhesion, Signal transduction, Skin

## Abstract

Impairment of desmosomal cell-cell adhesion leads to life-threatening diseases, such as the autoimmune skin-blistering disorder pemphigus vulgaris (PV). Disease management strategies that stabilize intercellular adhesion, in addition to the existing immunosuppression therapies, may result in improved clinical outcomes. Previous findings showed that the serine protease inhibitor SERPINB5 promotes intercellular adhesion by binding to and regulating the localization of the desmosomal adapter molecule desmoplakin (DSP) at the plasma membrane. We here show that SERPINB5 overexpression prevents PV-IgG–mediated loss of cell-cell adhesion and DSP dissociation from the cell membrane. We mechanistically demonstrate that SERPINB5 loss deregulates TGF-β signaling, a pathway known to destabilize DSP in keratinocytes. TGF-β signaling was also activated in skin biopsies of patients with PV and keratinocytes treated with PV autoantibodies, suggesting a contribution to disease. Inhibition of TGF-β signaling ameliorated PV-IgG–mediated loss of cell-cell adhesion, increased DSP membrane expression, and prevented PV-IgG–induced blister formation in a human ex vivo skin model. Together, SERPINB5 modulates DSP and intercellular adhesion through the regulation of TGF-β signaling. Further, TGF-β signaling was identified as a potential target for pemphigus treatment.

## Abstract 2

We show SERPINB5 modulates DSP and intercellular adhesion through the regulation of TGF-β signalling, which was identified as a potential target for pemphigus treatment.

## Introduction

The skin provides a barrier against external insults and is critical for organism homeostasis. Keratinocytes represent the main cell type within the epidermis, the epithelium of the skin. The epidermis is exposed to constantly changing mechanical stresses, and its integrity relies on strong connections of keratinocytes, which provide the necessary resilience against the acting forces (1). Desmosomes, due to their molecular arrangement and linkage to the intermediate filament (IF) cytoskeleton, are ideally suited to facilitate these robust intercellular adhesions (2, 3). Accordingly, desmosomes are abundant in tissues that are exposed to high mechanical forces, such as the epidermis or the myocardium (4). Desmosomes are made up of an intercellular core of the desmosomal cadherins desmoglein (DSG) 1–4 and desmocollin 1–3. These cadherins are coupled to IFs through intracellular adapter proteins, such as plakoglobin (PG), plakophilins, and desmoplakin (DSP) (3).

Dysfunction in desmosomal cell-cell adhesion leads to severe pathologies (5). Mutations of desmosomal proteins are directly associated with arrhythmogenic cardiomyopathy (6, 7). Further, autoantibodies targeting DSG1 and or DSG3 lead to the potentially fatal autoimmune skin-blistering diseases pemphigus vulgaris (PV) and pemphigus foliaceus (8–10). The microscopic hallmarks of pemphigus are intraepithelial blister formation and acantholysis, which is the loss of adhesion between keratinocytes. Mechanistically, it has been shown that autoantibodies induce steric hindrance, altered protein turnover, and aberrant signaling involving p38-MAPK, SRC, EGFR, and PKC, which all contribute to the disease outcome (11). Current treatment strategies for PV rely on immunosuppression strategies, such as corticosteroid administration, steroid-sparing immunosuppressive drugs (e.g., azathioprine and mycophenolate mofetil), and B cell depletion (12, 13), which are associated with profound side effects. Given the causal relationships between dysfunctional desmosomal adhesion and clinical phenotype, strategies that stabilize intercellular adhesion may serve as targeted therapeutic options. Thus, the exploitation of known mechanisms regulating membrane trafficking, expression, and turnover of desmosomal molecules may serve an unmet clinical need.

In the current study, we show that SERPINB5 rescues autoantibody-mediated loss of cell cohesion by enhancing DSP localization at the cell membrane. SERPINB5 is a nonclassical SERPIN, which is believed not to inhibit serine proteases (14). SERPINB5, also called MASPIN, was first identified as a tumor suppressor in breast cancer models. It was associated with migration and adhesion of cells (15). However, the underlying mechanisms are not well understood. It was previously shown that TGF-β signaling negatively modulates cell-cell adhesion and DSP expression in keratinocytes (16), and TGF-β in cooperation with p53 positively modulates SERPINB5 expression in mammary epithelial cells (17). We here identified TGF-β signaling as a mediator of cell-cell adhesion loss in PV patient samples. Inhibiting TGF-β activation prevented loss of intercellular adhesion in vitro and blister formation ex vivo in human skin.

## Results

### SERPINB5 rescues PV-mediated loss of intercellular adhesion.

**Figure 1:**
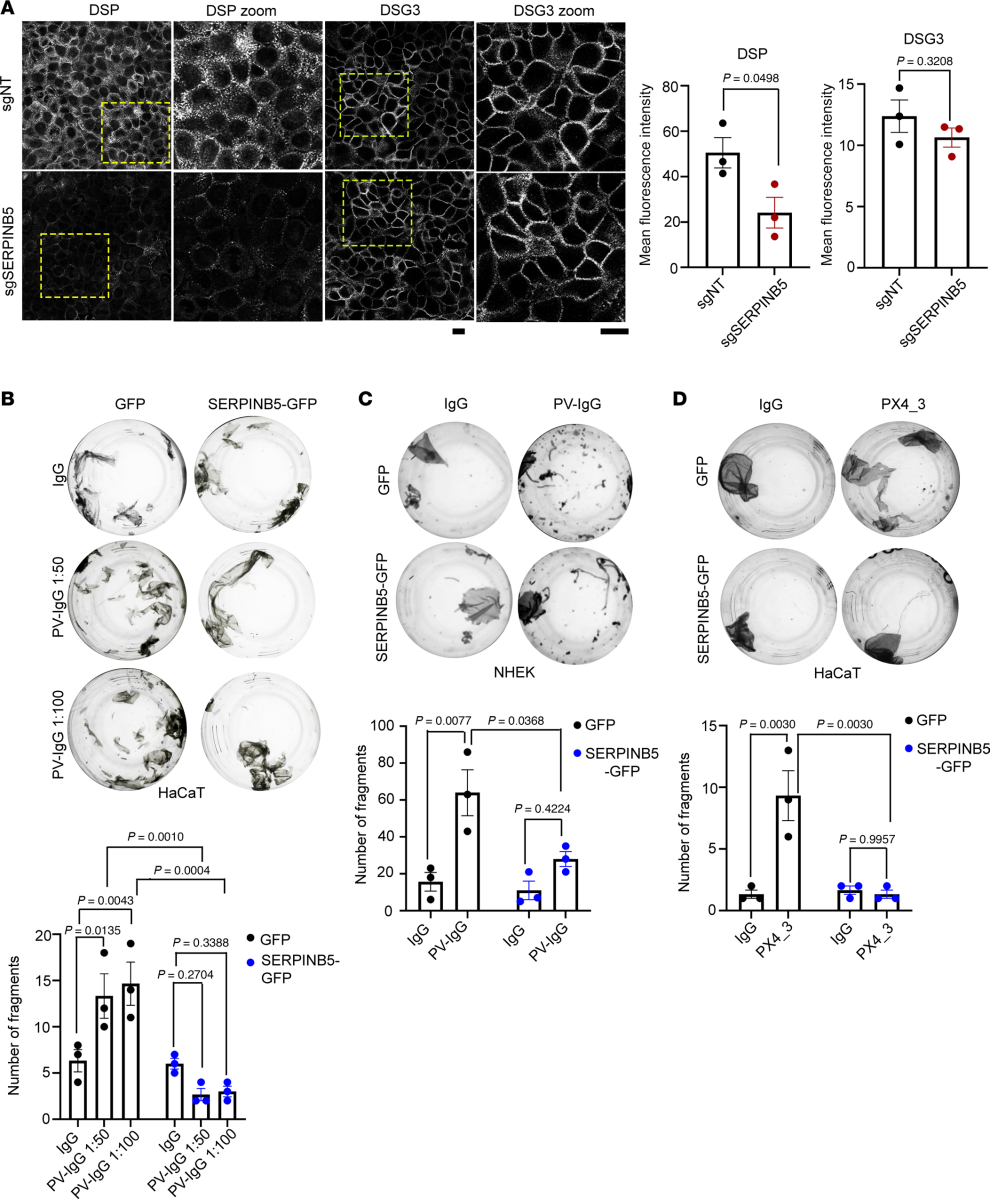
SERPINB5 ameliorates PV-IgG–mediated loss of intercellular adhesion. (**A**) Immunofluorescence staining of small guide RNA (sg)- Non Target (NT) control (sgNT) and sgSERPINB5 HaCaT cells using DSG3 and DSP antibodies. Scale bar, 10 μm. Quantification of DSP and DSG3 mean fluorescence intensity of 3 independent experiments shown. Each data point represents the average of at least 3 fields of view of 1 biological replicate. Unpaired Student’s 2-tailed *t* test used for statistical analysis. (**B**) Dispase-based dissociation assay of HaCaT cells overexpressing GFP or SERPINB5-GFP and treated with IgG or PV-IgG at indicated concentrations. Representative images and quantifications of *n* = 3 are shown. Two-way ANOVA, Šídák correction used for statistical analysis. (**C**) Dispase-based dissociation assay of NHEK cells overexpressing GFP or SERPINB5-GFP and treated with IgG or PV-IgG (1:100). Representative images and quantifications of *n* = 3 are shown. Two-way ANOVA, Tukey’s multiple comparison used for statistical analysis. (**D**) Dispase-based dissociation assay of HaCaT cells overexpressing GFP or SERPINB5-GFP and treated with IgG or PX4_3 (1:100). Representative images and quantifications of *n* = 3 are shown. Two-way ANOVA, Tukey’s multiple comparison used for statistical analysis.

Immunostaining-based analysis of DSP in SERPINB5-knockdown (SERPINB5-KD) Human adult high Calcium low Temperature (HaCaT) keratinocytes (Supplemental Figure 1A; supplemental material available online with this article; https://doi.org/10.1172/jci.insight.183024DS1) verified our recent finding (18) that SERPINB5 is a positive regulator of DSP membrane localization, while DSG3 localization and cytokeratin organization were unaltered (Figure 1A and Supplemental Figure 1B). This is also in line with our results that SERPINB5 is required for normal intercellular adhesive strength in keratinocytes (18). SERPINB5-mediated positive regulation of DSP is posttranslational, as the transcript levels of Dsp and Dsg3 were unchanged upon loss of SERPINB5 (Supplemental Figure 1C). To study the effect of SERPINB5 on PV-IgG–mediated loss of cell cohesion, we overexpressed SERPINB5 in keratinocytes (Supplemental Figure 1, D and E). Dissociation assays showed that overexpression of SERPINB5 rescued loss of cell-cell adhesion in HaCaT and primary human keratinocytes (NHEK cells) induced by incubation with autoantibody fractions of patients with PV (PV-IgG) (Figure 1, B and C). These findings were verified using PX4_3, a single chain variable fragment cloned from a PV patient B cell repertoire that targets both DSG3 and DSG1 (19) (Figure 1D).

### SERPINB5 stabilizes DSP at the cell membrane under PV-IgG conditions.

**Figure 2:**
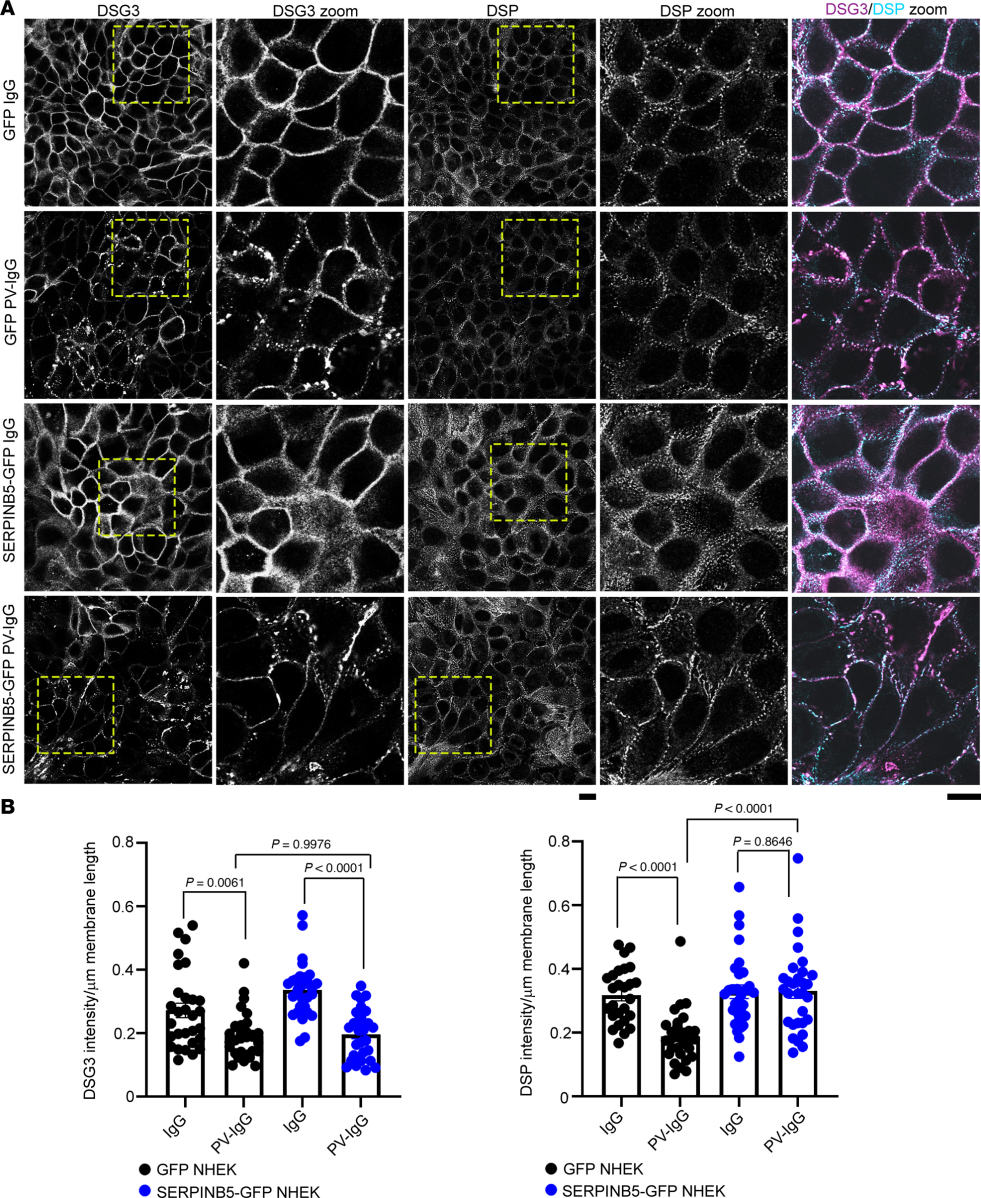
SERPINB5 prevents PV-IgG–mediated DSP rearrangement. (**A**) Immunofluorescence staining of DSG3 and DSP in NHEK cells expressing GFP or SERPINB5-GFP, respectively. Scale bar, 10 μm. (**B**) Quantification of DSG3 and DSP fluorescence intensity/membrane length of individual cells from 3 independent experiments shown. One-way ANOVA, Tukey’s multiple comparison used for statistical analysis.

Next, we analyzed the distribution of DSG3 in NHEK cells in the context of SERPINB5 overexpression. DSG3 staining showed the well-established fragmented pattern in the membrane and a generally reduced expression upon treatment with PV-IgG, which was unaltered in the presence of SERPINB5-GFP (Figure 2A and Supplemental Figure 2, A and B). However, staining of the central desmosome constituent DSP revealed that the reduction at the membrane and the trend toward reduced expression upon treatment with PV-IgG was prevented by SERPINB5 overexpression (Figure 2B and Supplemental Figure 2, A and B). Analysis of the abundance of other desmosomal proteins showed no significant alterations upon overexpression of SERPINB5. This suggests that, under PV-IgG exposure, SERPINB5 stabilizes DSP expression and localization largely independently of DSG3 and rescues loss of cell-cell adhesion.

### SERPINB5 regulates TGF-β signaling, which is activated in PV patient samples.

**Figure 3:**
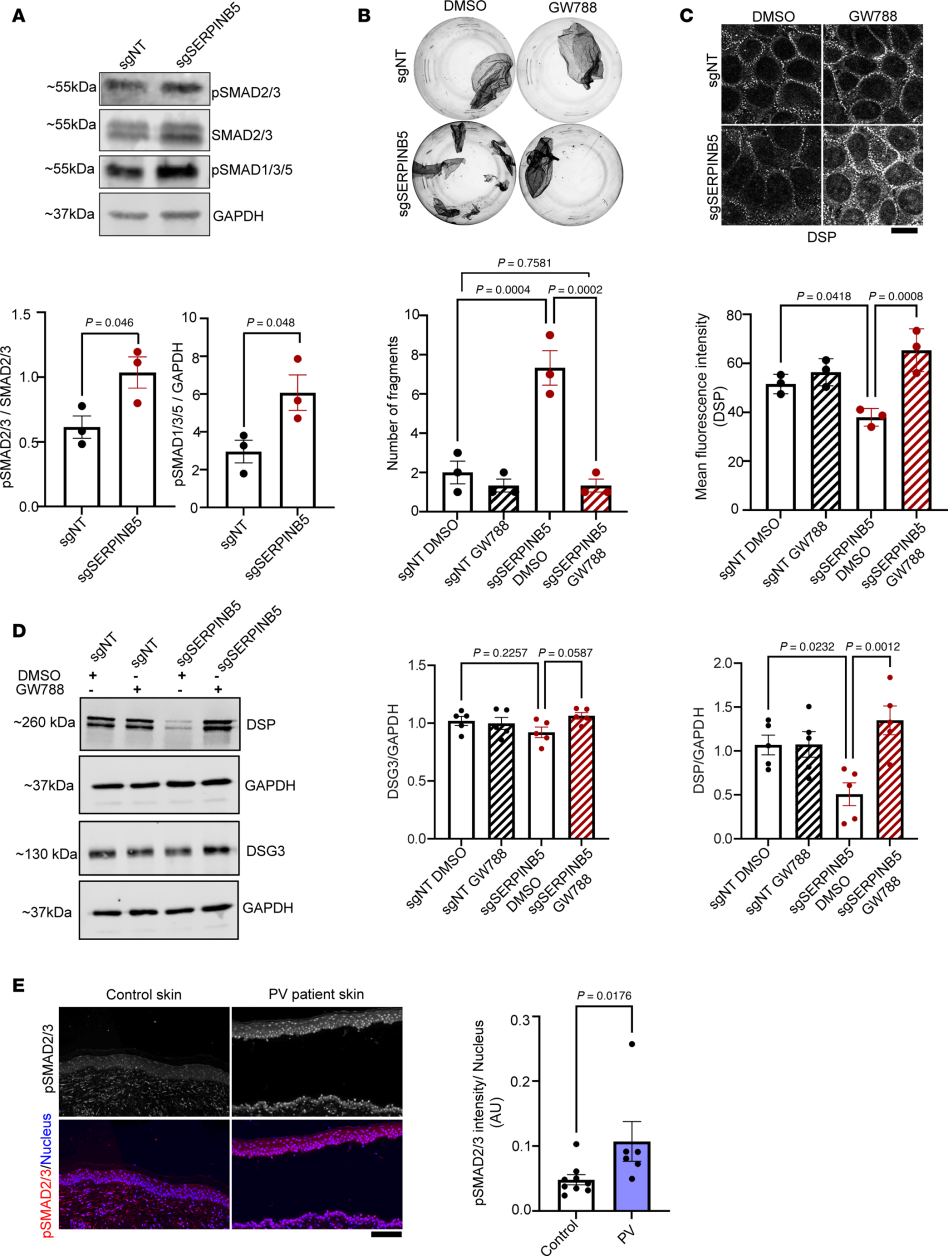
TGF-β signaling is elevated in PV patient samples and modulated by SERPINB5. (**A**) Western blot analysis of sgNT and sgSERPINB5 HaCaT cell lysates using p-SMAD2/3, SMAD2/3, p-SMAD1/3/5, and GAPDH antibodies. Representative Western blot images and quantifications of indicated proteins (*n* = 3) are shown. Unpaired Student’s 2-tailed *t* test was used for statistical analysis. (**B**) Dispase-based dissociation assay of HaCaT cells with sgNT or sgSERPINB5, in combination with either DMSO or GW788388 (GW788) for 24 hours. Representative images and quantifications of *n* = 3 are shown. One-way ANOVA, Šídák multiple comparison used for statistical analysis. (**C**) Immunofluorescence staining of DSP in HaCaT cells expressing sgNT or sgSERPINB5 treated with DMSO or GW788. Scale bar, 10 μm. Quantification of DSP mean fluorescence intensity of 3 independent experiments shown. Each data point represents the average of at least 3 fields of view of 1 biological replicate. One-way ANOVA, Tukey’s multiple comparison used for statistical analysis. (**D**) Western blot analysis of lysates from sgNT or sgSERPINB5 cells incubated with DMSO or GW788 for 24 hours, using DSP, DSG3, and GAPDH antibodies. Representative Western blot images and quantifications of indicated proteins (*n* = 5) are shown. One-way ANOVA, Šídák correction used for statistical analysis. (**E**) Immunofluorescence staining of p-SMAD2/3 in human epidermal biopsy sections from healthy controls or patients with PV. DAPI served to visualize nuclei. Scale bar, 10 μm. Quantification shows the mean intensity of p-SMAD2/3 per nucleus (*n* = 9 controls and *n* = 6 patients). Unpaired Student’s *t* test used for statistical analysis.

TGF-β signaling is a known negative regulator of DSP expression and cell-cell adhesion (16). Interestingly, KD of SERPINB5 in HaCaT keratinocytes led to significantly enhanced levels of phosphorylated (p-) SMAD2/3 and p-SMAD1/3/5 (Figure 3A). Inhibiting SMAD2/3 and SMAD1/3/5 signaling by the small molecule inhibitor GW788388 (GW788) (Supplemental Figure 3A) rescued loss of cell-cell adhesion (Figure 3B), and the reduction of DSP amount and membrane localization (Figure 3, C and D) associated with SERPINB5 KD. DSG3 expression remained unaltered under these conditions. Phosphorylation at S665 was suggested as a proadhesive modification in PG (20, 21). We evaluated the role of TGF-β in modulating PG localization and phosphorylation. PG membrane localization, which was prevented by inhibition of TGF-β signaling, was reduced upon KD of SERPINB5 (Supplemental Figure 3B). However, the phosphorylation and expression of PG remained unaltered (Supplemental Figure 3C), demonstrating that the effect of TGF-β primarily affects membrane localization but is independent of total PG levels and S665 phosphorylation state.

Given that DSP localization at the membrane was impaired both in SERPINB5-KD cells and in keratinocytes incubated with PV-IgG, we evaluated changes in TGF-β signaling in PV patient biopsies. Indeed, analysis of 9 healthy control and 6 PV patient samples revealed enhanced levels of p-SMAD2/3 in PV epidermis (Figure 3E). Furthermore, keratinocytes treated with PV-IgG and PX4_3 showed elevated p-SMAD2/3 and p-SMAD1/3/5 levels (Supplemental Figure 3D), indicating that PV-IgG activates TGF-β signaling. Based on our observation of SERPINB5-mediated suppression of TGF-β signaling, we tested whether PV-IgG modulates SERPINB5 levels. Indeed, SERPINB5 staining intensity was reduced upon PV-IgG treatment in cultured keratinocytes and PV patient samples (Supplemental Figure 3, E and F), which was further supported by Western blot showing a trend toward reduced protein levels (Supplemental Figure 3G). Together, this supports the hypothesis that SERPINB5 downregulation in response to PV-IgG is a mediator of elevated TGF-β signaling.

### TGF-β inhibition ameliorates PV-IgG–mediated cell dissociation in cultured keratinocytes and acantholysis in an ex vivo human skin model.

**Figure 4:**
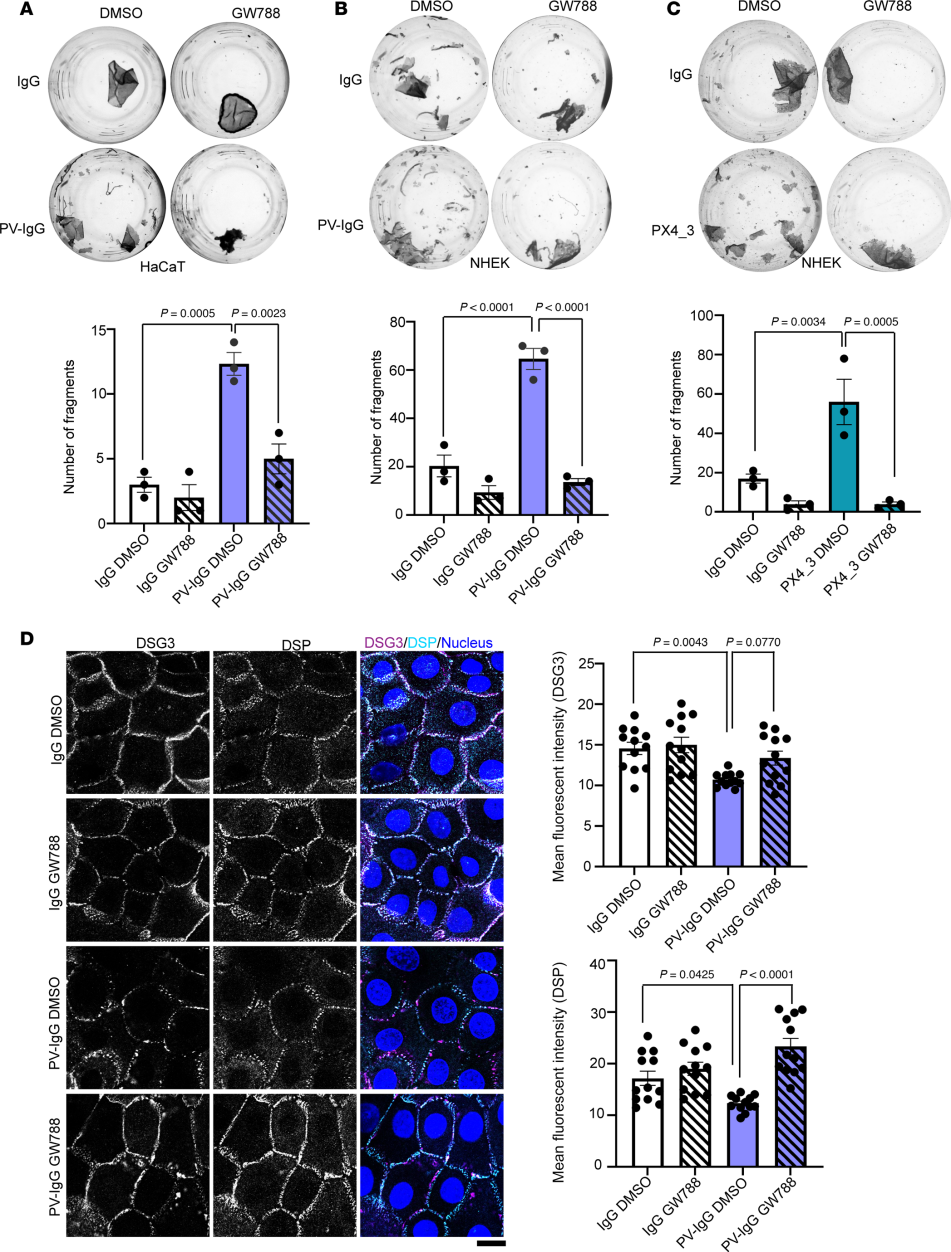
Inhibition of TGF-β signaling rescues PV-IgG–mediated loss of cell-cell adhesion. (**A**) Dispase-based dissociation assay of HaCaT cells treated with IgG or PV-IgG, in combination with either DMSO or GW788 for 24 hours. Representative images and quantifications of *n* = 3 are shown. One-way ANOVA, Tukey’s multiple comparison used for statistical analysis. (**B**) Dispase-based dissociation assay of NHEK cells treated with IgG or PV-IgG, in combination with either DMSO or GW788, for 24 hours. Representative images and quantifications of *n* = 3 are shown. One-way ANOVA, Tukey’s multiple comparison used for statistical analysis. (**C**) Dispase-based dissociation assay of NHEK cells treated with IgG or PX4_3 in combination with either DMSO or GW788 for 24 hours. Representative images and quantifications of *n* = 3 are shown. One-way ANOVA, Tukey’s multiple comparison used for statistical analysis. (**D**) Immunofluorescence staining of DSP and DSG3 in NHEK cells treated with IgG or PV-IgG, in combination with either DMSO or GW788 for 24 hours. DAPI served to visualize nuclei. Scale bar, 10 μm. Quantification of DSG3 and DSP mean fluorescence intensity from different areas of 3 independent biological replicates shown. One-way ANOVA, Tukey’s multiple comparison used for statistical analysis.

Our findings suggest TGF-β signaling as a mechanism contributing to loss of cell-cell adhesion in PV. To address this, we inhibited the activation of SMAD2/3 and SMAD1/3/5 by GW788. Coincubation of either HaCaT keratinocytes or NHEKs together with PV-IgG and GW788 prevented loss of cell-cell adhesion (Figure 4, A and B). Similarly, PX4_3-mediated loss of cell-cell adhesion was blocked by GW788 treatment (Figure 4C). Further, inhibition of TGF-β signaling significantly increased DSP localization at the cell membrane in both IgG control– and PV-IgG–treated NHEK cells, while DSG3 localization at the cell membrane was slightly enhanced (Figure 4D). DSP and DSG3 protein levels were reduced upon treatment with PV-IgG, which was prevented by GW788 (Supplemental Figure 4A). SERPINB5 levels were largely unchanged by GW788 treatment (Supplemental Figure 4B), suggesting that TGF-β signaling does not modulate SERPINB5 expression. However, PV-IgG–mediated increase in p-SMAD2/3 phosphorylation was absent in cells overexpressing SERPINB5 (Supplemental Figure 4C), suggesting SERPINB5 is an upstream regulator of TGF-β signaling.

**Figure 5:**
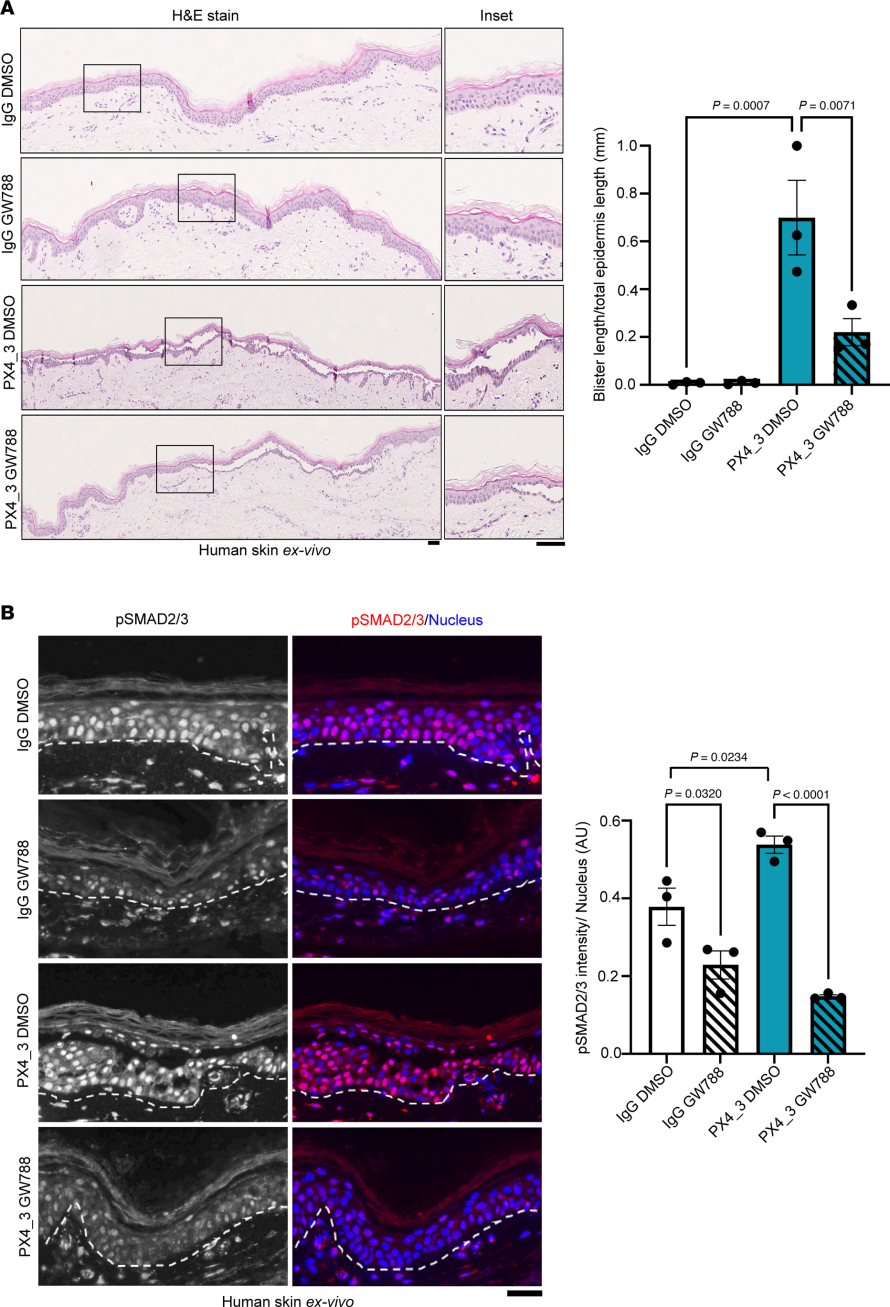
TGF-β inhibition rescues PV-IgG–mediated acantholysis. (**A**) H&E staining of human skin explants injected with IgG or pX4_3 and DMSO/GW788, respectively. Scale bar, 10 μm. Bar graphs represent quantification of blister length versus total epidermis length; each dot represents independent biological replicates (*n* = 3). One-way ANOVA, Šídák correction used for statistical analysis. (**B**) Immunofluorescence staining of human skin sections treated with Ctrl-IgG or pX4_3 and DMSO/GW788 using p-SMAD2/3 (red/white) antibodies and DAPI to stain nucleus (blue). Representative images are shown. Scale bar, 10 μm. Quantification showing the mean intensity of p-SMAD2/3 normalized to the respective number of nuclei within each experiment (*n* = 3); each data point represents 1 biological replicate. One-way ANOVA, Šídák correction used for statistical analysis.

We finally tested if inhibition of TGF-β influences blister formation in the skin. To do so, we applied a passive transfer human ex vivo model to study the effects on blistering phenotypes of the disease. Human skin explants were injected subcutaneously with 40 μg of purified pX4_3 or IgG as a control. PX4_3 induced intraepidermal blistering after 24 hours, which was significantly reduced by concomitant injection of GW788, suggesting that TGF-β inhibition ameliorates acantholysis in human skin (Figure 5A). SMAD2/3 inhibition in these tissues was verified by immunostainings (Figure 5B). Further, activation of SMAD2/3 was also seen under PX4_3 injections in human ex vivo skin, further supporting that PV-IgG activates TGF-β signaling.

## Discussion

### SERPINB5 regulates DSP localization at the cell membrane and increases intercellular adhesive strength.

In our previous study, we identified SERPINB5 as an interacting partner and regulatory molecule of DSP in keratinocytes (18). In the current study, we were interested in understanding the relevance of the SERPINB5/DSP axis in a disease context with impaired adhesion. PV is an autoimmune disease with autoantibody development against the desmosomal cadherins DSG1 and DSG3, resulting in blister formation in the skin and oral mucosa. It is established that autoantibodies cause loss of cell-cell adhesion by altered turnover and stability of DSG3 and DSG1 through signaling-dependent and -independent mechanisms (11). Methods to stabilize cell-cell adhesion may represent a tailored approach to ameliorate the adverse effects of the autoantibodies from patients with PV. For example, it has been shown that overexpression of DSG3 in NHEKs prevents PV-IgG–induced loss of cell-cell adhesion (22). In a recent study, it was demonstrated that the phosphodiesterase 4 inhibitor apremilast ameliorated PV-IgG–induced blisters and cell-cell adhesion. Mechanistically, apremilast inhibited keratin retraction and promoted DSP stability through phosphorylation of PG at S665, which has previously been shown to be proadhesive (20, 21). In our study, we asked if SERPINB5-mediated regulation of DSP could, in a similar manner, improve the PV-IgG–mediated loss of cell-cell adhesion. Dispase-based cell cohesion assay showed SERPINB5-GFP overexpression in NHEK cells and HaCaT keratinocytes rescued PV-IgG–mediated loss of cell-cell adhesion and prevented loss of DSP membrane localization under PV-IgG treatment. SERPINB5 KD reduced DSP protein levels; however, no change in mRNA levels was observed, indicating SERPINB5 mediates stabilization of DSP levels at the cell junctions. Interestingly, SERPINB5 did not alter membrane localization of DSG3 or the levels of other desmosomal molecules under these conditions. demonstrating that the effect is restricted to DSP. This is similar to the study using apremilast, in which fragmentation of DSG3 staining was unaltered in response to apremilast, though loss of adhesion was blocked through apremilast-mediated increase in DSP. Further, apremilast restored PV-IgG–mediated defects in keratin cytoskeleton organization (21). It is interesting to note that modulating specific constituents of the desmosome (i.e., the transmembrane adhesion molecules, plaque proteins, and intermediate filament insertion) individually is sufficient to promote intercellular adhesion. So far it is unclear whether exclusive mechanisms are in place or whether these factors act interdependently, finally resulting in increased intercellular adhesion. Still, it is interesting to speculate that these individual targets can be exploited to precisely tune adhesion as a therapeutic principle in disease.

Our study also implicates TGF-β in the SERPINB5-dependent regulation of DSP. This is based on previous observations suggesting SERPINB5 as a negative modulator of TGF-β signaling (23). Interestingly, TGF-β signaling was also determined to be a negative regulator of DSP localization at the cell membrane (16). Analysis of PV patient samples showed a higher activity of SMAD2/3, a downstream effector of the TGF-β pathway. In line with patient data, treatment of keratinocytes with PV-IgG or PX4_3 enhanced the activation of SMAD2/3, and inhibition of TGF-β activation increased DSP levels at the cell membrane under control and PV-IgG conditions. This suggests that SERPINB5 through dampening the TGF-β pathway increases DSP localization at the cell membrane, resulting in increased adhesive strength.

### Targeting TGF-β signaling as a therapeutic approach in PV.

PV impairs patients’ quality of life dramatically and can lead to extreme reduction of general health status. Therapeutic strategies for PV include corticosteroids, steroid-sparing drugs, and B cell suppression therapies (24) or plasmapheresis (12, 13). Autoantibodies induce signaling pathways, such as p38-MAPK, and inhibition of these pathways has been tested in mouse and human skin models. More directed approaches to deplete the DSG3-specific B cells are now being investigated; for example, chimeric antigen receptor T cell therapy holds great promise and is the subject of a clinical trial (25). However, due to disease heterogeneity, genetic diversity, and therapy resistance, it is still important to investigate new therapeutic targets. Targeted modulation of cell-cell adhesion may serve as a supplementary measure for better management of the disease.

As we have observed TGF-β activation in PV patient samples and PV-IgG/PX4_3–treated keratinocytes, we inhibited TGF-β activation using the small molecule inhibitor GW788. Importantly, inhibition of TGF-β ameliorated PV-IgG– and PX4_3-mediated loss of cell-cell adhesion and blister formation in an ex vivo passive-transfer model of PV. GW788 treatment also rescued the loss of DSP from the cell-cell membrane and stabilized DSG3. This indicates that, although the link between SERPINB5 and TGF-β signaling promoted mainly DSP at the membrane, directly modulating TGF-β signaling appeared to have additional effects on desmosomal molecules. It is possible that the direct interaction of SERPINB5 with DSP (18) is related to this phenomenon. Nevertheless, in line with our findings of SERPINB5 as a negative modulator of TGF-β activation, PV-IgG treatment in keratinocytes and PV patient skin biopsies showed reduced expression of SERPINB5, suggesting TGF-β activation in response to PV-IgG is at least in part regulated by SERPINB5. The mechanisms by which PV-IgG activates TGF-β signaling need further investigation.

TGF-β activation has been associated with other skin disorders, such as psoriasis and epidermolysis bullosa, and squamous cell carcinoma (26–28). TGF-β inhibition through topical application of small molecule inhibitors has been demonstrated as a therapeutic option in psoriasis, as systemic use of TGF-β inhibitors can lead to severe side effects because of pleiotropic roles of TGF-β in development and homeostasis (29). Several ways of targeting TGF-β signaling by inhibitors of downstream effector molecules have made it possible to reduce side effects. Many of these inhibitors are in clinical trials for cancer treatments (30). Thus, a TGF-β inhibition strategy should be carefully designed for use in PV, and testing the effectivity for a topical use should be considered.

In conclusion, we identify SERPINB5 as a modulator of DSP membrane localization through TGF-β signaling. Further, elevated TGF-β activation was observed in PV patient samples, the inhibition of which may serve as an option to improve acantholysis in PV.

## Methods

### Sex as a biological variable.

The findings were not biased based on sex and could be applicable to both sexes, however this study did not do a detailed sex-based analysis.

### Cell culture and generation of lentiviral constructs and stable cell lines.

Spontaneously immortalized HaCaT keratinocytes (31) were cultured in a humidified atmosphere of 5% CO_2_ and 37°C in DMEM (Sigma-Aldrich, D6546) containing 1.8 mM Ca^2+^ and complemented with 10% fetal bovine serum (Merck, S0615), 50 U/mL penicillin (VWR, A1837.0025, D6546), 50 μg/mL streptomycin (VWR, A1852.0100), and 4 mM l-glutamine (Sigma-Aldrich, G7513). Lentiviral particles were generated according to standard procedures. HEK293T cells (a gift from J. Schwaller laboratory, Department of Biomedicine, University of Basel, Basel, Switzerland) were transfected with the lentiviral packaging vector psPAX2 (12259, Addgene), the envelope vector pMD2.G (12260, Addgene), and the respective construct plasmid using TurboFect (Thermo Fisher Scientific). At 48 hours posttransfection, virus-containing supernatant was collected and concentrated using Lenti-Concentrator (OriGene), for at least 2 hours at 4°C. Cells were transduced with the respective virus particles in an equal ratio using 5 μg/mL polybrene (Sigma-Aldrich) according to the manufacturer’s instructions. Twenty-four hours posttransduction for HaCaT keratinocytes and 8 hours later for primary human keratinocytes, medium was exchanged and puromycin selection was applied. Cells were cultivated for at least 1 week under selective pressure, before starting with the respective experiments. Expression of the respective construct was verified via Western blot analysis. The cloning of constructs for SERPINB5 overexpression, sgSERPINB5, and sgNT is described in the publication (23).

### Isolation of NHEK cells.

Foreskin tissue was obtained during circumcision of patients (Department of Urology, University Hospital Basel). The skin samples were washed with PBS containing 300 U/mL of penicillin (A1837, AppliChem), 300 U/mL of streptomycin sulfate (A1852, AppliChem), and 7.5 μg/mL of amphotericin B (A2942 Sigma-Aldrich). The dermis and epidermis were separated using 5 mg/mL Dispase II solution (D4693, Sigma-Aldrich) in HBSS (H8264, Sigma-Aldrich) containing 300 U/mL penicillin, 300 U/mL streptomycin sulfate, and 2.5 μg/mL amphotericin B. The detached epidermis was digested with TryplE dissociation reagent (12605028, Gibco) containing 100 U/mL penicillin and 100 U/mL streptomycin sulfate at 37°C for 20 minutes. Following the digestion, keratinocytes were isolated by passing through a 70 μm cell filter (431751, Corning). The isolated NHEK cells were then seeded at a density of ~8 × 10^4^ cells/cm^2^ in EpiLife medium containing 60 μmol/L CaCl_2_ (MEPI500CA, Gibco), 1% human keratinocyte supplement (S0015, Gibco), 1% penicillin/streptomycin, and 2.5 μg/mL amphotericin B. After 3 days, the medium was changed and amphotericin B was discontinued.

### Dispase-based dissociation assay.

HaCaT cells were seeded in 24-well plates until 100% confluence and treated with IgG, PV-IgG, or PX4_3 and drug inhibitor as indicated. The NHEK cells were seeded in a 24-well plate and grown to confluence, followed by addition of 1.2 mmol/L CaCl_2_ for 24 hours to induce differentiation. The cells were then washed with PBS and incubated with 250 μL Dispase II (Sigma-Aldrich, D4693) solution (50 mg in 10 mL HBSS) for 30 minutes at 37°C, for detachment of the cell monolayer. A total of 150 μL of HBSS (Huber Lab, A3140) was added to the floating monolayer. The monolayer was subjected to homogeneous shear stress applied by 10× electrical pipetting with Eppendorf Xplorer 1000 (350 μL, L49475G). The fragments were documented and quantified with a stereomicroscope (Olympus, SZX2-TR30) with an attached camera (Canon, EOS 800D).

### Western blot.

Confluent cell monolayers were lysed with SDS lysis buffer (25 mM HEPES, 2 mM EDTA, 25 mM NaF, 1% SDS, pH 7.6) supplemented with an equal volume of a protease inhibitor cocktail (cOmplete, Roche Diagnostics). Lysates were sonicated and the total protein amount was determined with a BCA protein assay kit (Thermo Fisher Scientific) according to the manufacturer’s instructions. The proteins were denatured by heating in Laemmli buffer, for 10 minutes at 95°C. Membranes were blocked in Odyssey blocking buffer (Li-Cor) for 1 hour at room temperature (RT). The following primary antibodies were diluted with Odyssey blocking buffer in TBS containing 0.1% Tween 20 (Thermo Fisher Scientific) and incubated overnight at 4°C, with rotation: rabbit SERPINB5 (MASPIN ab182785 Abcam), mouse GAPDH mAb (clone 0411, sc-47724 Santa Cruz Biotechnology), rabbit pSMAD2/3 (AP0548 Lubioscience), rabbit pSMAD1/3/5 (ab95455 Abcam), mouse SMAD2/3 (sc-133098 Santa Cruz Biotechnology), and pPG S665 (from Jens Waschke, Ludwig-Maximilians-University [LMU], Munich, Germany). Goat anti-mouse 800CW and goat anti-rabbit 680RD (925-32210 and 925-68071, both Li-Cor) were used as secondary antibodies, incubated for 1 hour at RT. Odyssey FC imaging system was used for imaging the blots, and band intensity was quantified with Image Studio (both Li-Cor). The blots were internally normalized to the respective means of individual proteins for every biological replicate.

### Immunofluorescence staining and imaging.

Cell were grown on coverslips till confluence and were fixed with chilled methanol (at –20°C) for 10 minutes at 4°C. Blocking was done using 3% BSA and 1% normal goat serum in PBS for 1 hour at RT. The coverslips were incubated overnight at 4°C with the following primary antibodies: anti–DSG3-mAb (Invitrogen, 326300) and anti–DSP-mAb (NW39, from Kathleen Green, Northwestern University, Chicago, Illinois, USA). Cells were then washed 3 times with 1× PBS and incubated with the secondary Alexa Fluor–coupled antibodies (Thermo Fisher Scientific, A-11008, A-11004) for 1 hour at RT. DAPI (Sigma-Aldrich, D9542) was added for 10 minutes to counterstain nuclei. The coverslips were washed 3 more times with PBS and mounted using ProLong Diamond Antifade (Thermo Fisher Scientific, P36961). Images were taken with an HC PL APO CS2 63×/1.40 oil objective on a Stellaris 8 Falcon confocal microscope (Leica).

### Passive transfer ex vivo skin model.

For the ex vivo skin model, human skin pieces (approximately 1 cm^2^) were collected from the thigh of body donors having no history of skin disease, within 24 hours of decease. Skin at this time and after additional 24 hours of ex vivo incubation is still viable (32). Skin pieces were injected superficially with 40 μg of pX4_3 or 40 μg of control IgG in PBS in combination with DMSO and GW788 (Sigma-Aldrich, SML0116). These pieces were then incubated floating on DMEM (Sigma-Aldrich, D6546) including 10% FCS, 0.2% glutamate, and 0.5% penicillin/streptomycin for 24 hours. After 24 hours of incubation, a constant shear stress (10× 90° rotations with a custom-made rubber stencil) was applied, and the tissues were fixed in 4% paraformaldehyde for 24 hours. The specimens were then subjected to tissue processing, embedding in paraffin blocks, and subsequent histological examination.

### PV patient material.

Skin punch biopsies were taken from patients with PV as part of the diagnostic procedures by the Department of Dermatology, Universitätsklinikum Giessen Marburg. The control sections were taken from excess normal skin removed during the resection of skin tumors (Supplemental Table 1). These tissues were then embedded in paraffin blocks and sectioned using a microtome (Thermo Fisher Scientific, HM355S). The ELISA values from PV patient sera were 1,207 U/mL for DSG1 and 3,906 U/mL for DSG3.

### Histology and immunostaining of tissue sections.

Paraffin blocks were cut into 5 μm–thick sections using a microtome. H&E staining was carried out according to standard procedures. Briefly, the sections were stained for 5 minutes with Mayer’s hemalum solution (Sigma-Aldrich, 1.09249.1022), followed by washing, and then dehydrated using an increasing ethanol series and counterstained with 0.5% (w/v) eosin solution for 5 minutes. Following washing steps in ethanol and methyl salicylate, the sections were covered with DPX mounting medium (Sigma-Aldrich, 06522). For immunostaining, the sections were deparaffinized, and antigen retrieval was performed in citrate buffer (10 mM citric acid monohydrate [20276.235, VWR], pH6; 0.1% Triton X-100) for 20 minutes at 95°C. Permeabilization was done using 0.1% Triton X-100 in PBS for 5 minutes and then blocked with 3% BSA/0.12% normal goat serum in PBS for 1 hour at room temperature. The sections were incubated with the rabbit pSMAD2/3 (AP0548 Lubioscience) in PBS overnight at 4°C. After washing, the secondary antibodies were added for 1 hour at RT. DAPI (Sigma-Aldrich, D9542) was added for 10 minutes to stain the nuclei. Samples were embedded with Fluoromount Aqueous Mounting Medium (Sigma-Aldrich, F4680).

### Image analysis.

The figures were created with the use of Photoshop CC and Illustrator CC (Adobe). Immunofluorescence staining of cells was analyzed with ImageJ software (NIH) for quantifying DSG3, DSP, and p-SMAD2/3 mean intensity. Immunofluorescence staining of tissue sections was analyzed with QuPath-0.4.3. Cell nuclei were detected using DAPI, and then the intensity of the protein of interest was determined for each nucleus. H&E-stained and immunostained samples were scanned with a slide scanner (NanoZoomerS60, Hamamatsu). Blister and total length of each H&E-stained sample was measured with NDPview.2 (Hamamatsu).

### Statistics.

Statistical analysis was carried out using GraphPad Prism 8 software. Data sets were first tested for normal distribution using the Shapiro-Wilk normality test. Student’s 2-tailed *t* tests to compare 2 data sets and 1-way or 2-way ANOVA test for more than 2 data sets were performed to determine statistical significance (*P* < 0.05). Error bars in all graphs are presented as ± SEM. A minimum of 3 independent biological replicates were used for each experiment. *P* < 0.05 was considered statistically significant.

### Study approval.

The foreskin tissue from patients for isolation of primary keratinocytes was obtained after receipt of informed consent in accordance with the local ethics committee (EKNZ; date of approval: 11.06.2018, project ID: 2018-00963). The usage of patient material for this study was according to the Declaration of Helsinki and was approved by the Ethics Commission of the Medical Faculty at the University of Marburg under the number 169/19. For passive transfer ex vivo skin models, written informed consent was obtained from donors and next of kin for use of tissue samples for research.

### Data availability.

All data supporting the findings of this study are available within the paper and its supplement. Raw data and supporting data values for this manuscript are provided as a Supporting Data Values file.

## Author contributions

Conceptualization was done by VS and MR. Data acquisition was done by MR, MP, AZ, MM, TG, and HF. Data analysis was done by MR, MP, and HF. Funding was acquired by VS. Project administration was done by VS and MR. Resources were provided by VS, TC, DD, MH, and ES. Supervision was done by VS. Writing and preparation of the original draft were done by MR and VS. Review and editing of the manuscript were done by VS, MR, MP, AZ, HF, TC, DD, MH, and ES.

## Funding support

- Swiss National Science Foundation (197764 to VS).
- Novartis Foundation for Biomedical Research (to VS).

## Supplementary Material

Supplemental data

Unedited blot and gel images

Supporting data values

## Version 1 10/02/2025

In-Press Preview

## Version 2 11/24/2025

Electronic publication
