# Supplementary material for: SERPINB5/TGF-β signaling modulates desmoplakin membrane localization and ameliorates pemphigus vulgaris skin blistering
Source: JCI Insight. 2025 Oct 2;10(22):e183024. doi: 10.1172/jci.insight.183024 (PMC12643518; doi:10.1172/jci.insight.183024)
Supplement: Supplemental data [file jciinsight-10-183024-s006.pdf]

Supplementary Figure 1 (S1):

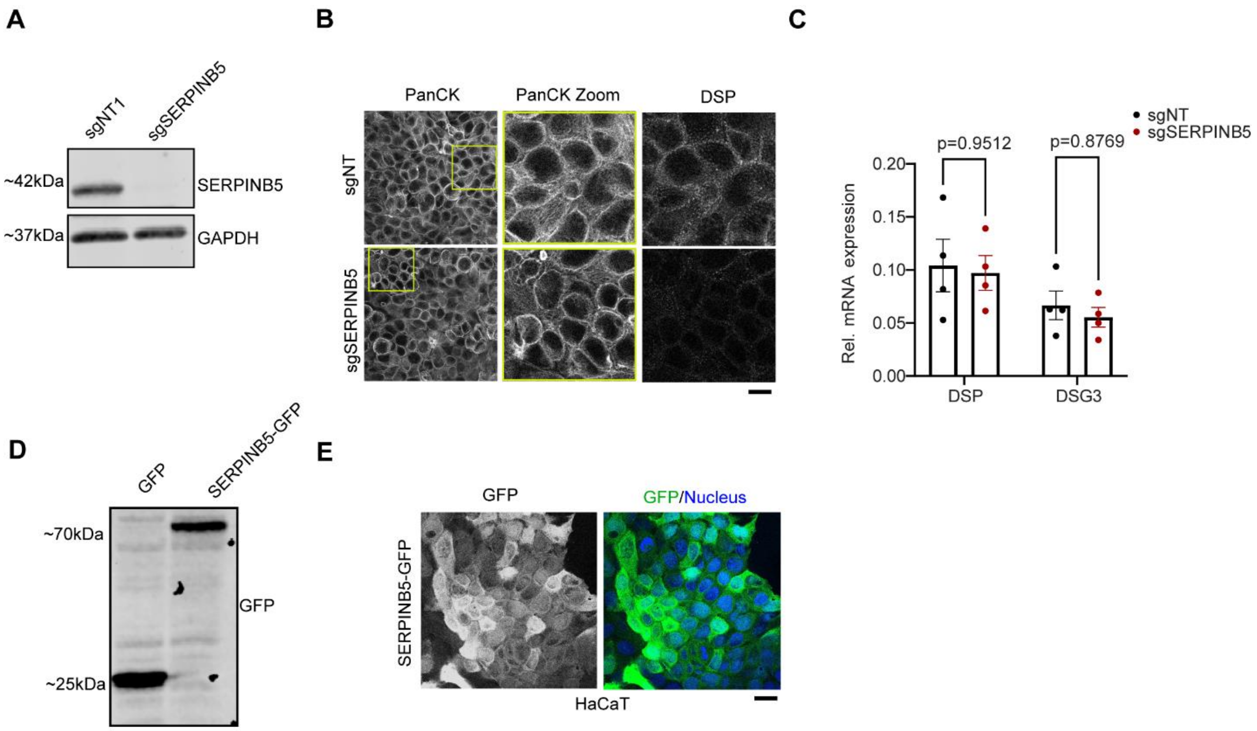

Figure S1A) Western blot analysis of sgNT and sgSERPINB5 HaCaT cell lysates using SERPINB5 and GAPDH antibodies to verify SERPINB5 knockdown. Representative Western blot images of 3 independent experiments are shown. **B)** Immunofluorescence image of pan cytokeratin (panCK) and DSP staining in sgNT and sgSERPINB5 cells. Representative image from 3 independent biological replicates. Scale bar = 10 μm. **C)** Relative mRNA expression of Dsp and Dsg3, normalized to Gapdh from sgNT and sgSERPINB5 cells. Each dot indicates independent biological replicates. Two-way-ANOVA, Sidak correction used for statistical analysis. **D)** Western blot image showing the expression of GFP from GFP control and SERPINB5-GFP overexpressing HaCaT cells. Representative image of 3 independent biological experiments shown. **E)** Immunofluorescence image of GFP from SERPINB5-GFP cells, to verify the expression. Green shows GFP signal and nucleus stained blue with DAPI. Scale bar = 10 μm.

Supplementary Figure 2 (S2):

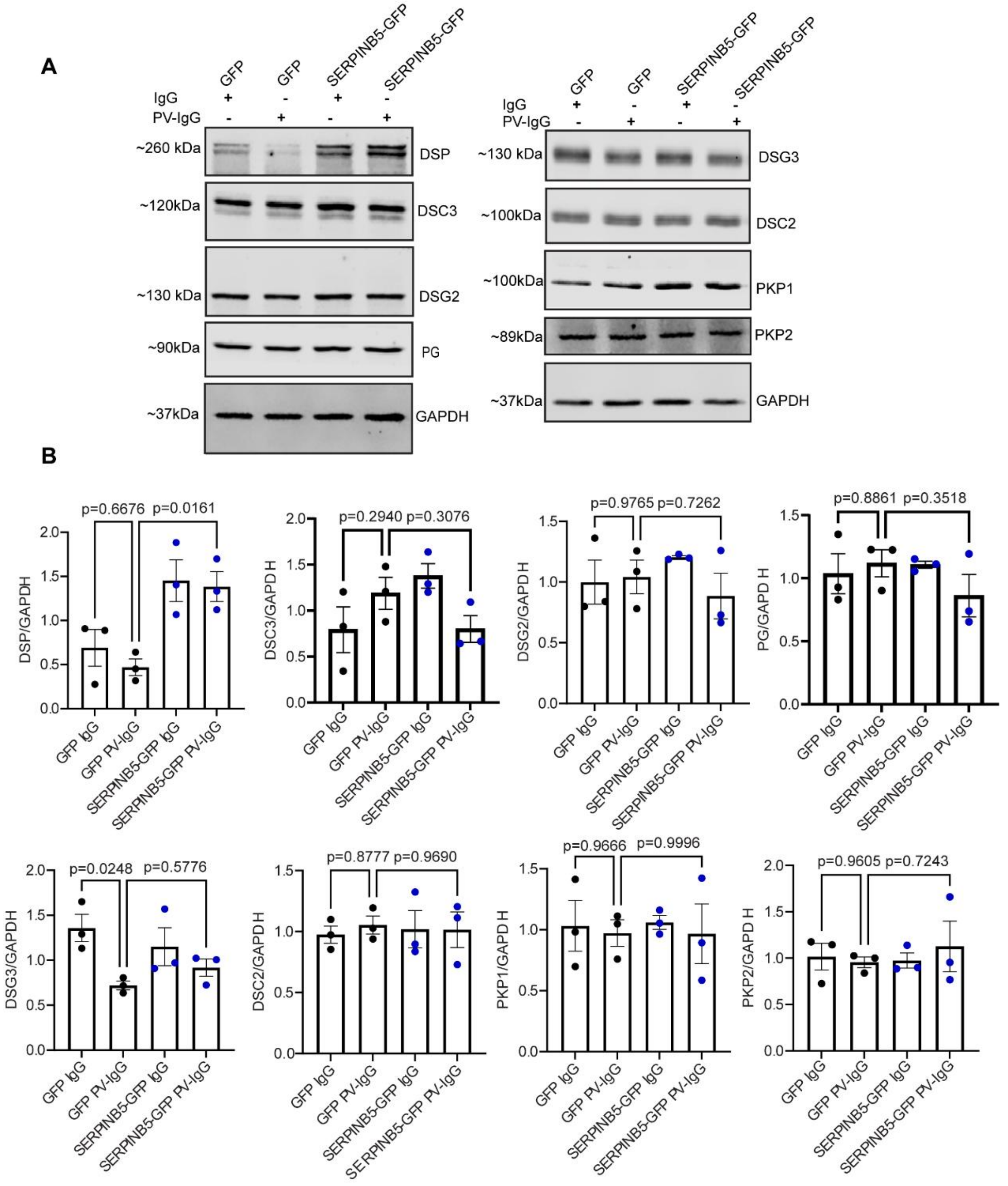

Figure S2A) Western blot images of GFP and SERPINB5-GFP HaCaT cell lysates using antibodies for indicated desmosomal proteins and GAPDH as loading control. Representative Western blot images of 3 independent experiments are shown. **B)** Quantifications of indicated proteins (n=3) are shown. One-way-ANOVA, Sidak correction used for statistical analysis.

Supplementary Figure 3 (S3):

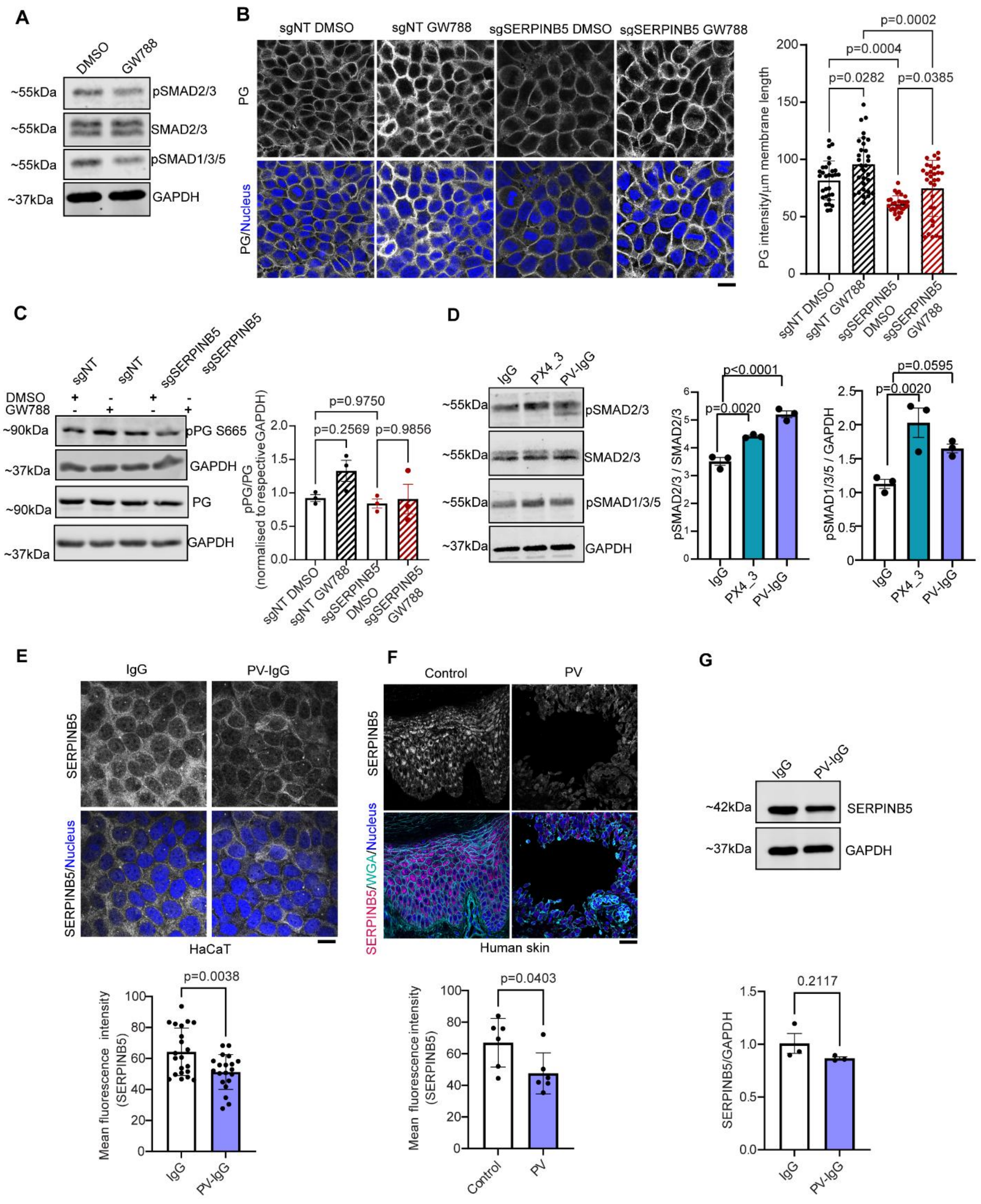

Figure S3A) Western blot analysis of HaCaT cell lysates treated with DMSO, GW788388 for 24 hours using pSMAD2/3, SMAD2/3, pSMAD1/3/5 and GAPDH antibodies. Representative Western blot images from 3 biological replicates are shown. GAPDH used as loading control. **B)** Immunofluorescence staining of plakoglobin (PG) in sgNT, sgSERPINB5 cells treated with DMSO and GW788 respectively. Scale bar = 10 μm. Graph showing quantification of PG fluorescence intensity/membrane length of individual cells from 3 independent experiments are shown. One-way-ANOVA, Tukey's multiple comparison used for statistical analysis. **C)** Western blot analysis of PG phosphorylation (pPG S665) in sgNT, sgSERPINB5 cells treated with DMSO and GW788. Quantitation showing pPG/PG levels relative to respective GAPDH values (n=3). One-way-ANOVA, Tukey's multiple comparison used for statistical analysis. **D)** Western blot analysis of HaCaT lysates from cells incubated with IgG, PX4_3 or PV-IgG for 24 hours, using pSMAD2/3, SMAD2/3, pSMAD1/3/5 and GAPDH antibodies. Representative Western blot images and quantifications of indicated proteins (n=3) are shown. One-way-ANOVA, Dunnett's correction used for statistical analysis. **E)** Immunofluorescence staining of HaCaT cells treated with IgG and PV-IgG for 24 hours, using SERPINB5 antibodies. Scale bar = 10 μm. Quantification of SERPINB5 mean fluorescence intensity of individual cells from 3 independent experiments are shown. Unpaired Students t-test used for statistical analysis. **F)** Immunofluorescence staining of SERPINB5 from human epidermal biopsy sections from healthy controls or PV patients. DAPI served to visualize nuclei. WGA used as membrane marker to visualize cell shape. Scale bar = 20 μm. Quantification shows the mean intensity of SERPINB5 (n=6 controls and n=6 patients). Unpaired Students t-test used for statistical analysis. **G)** Western blot image and quantification showing SERPINB5 expression in HaCaT cells treated with control IgG, PV IgG. Representative image of 3 independent biological replicates. Unpaired Students t-test used for statistical analysis.

Supplementary Figure 4 (S4):

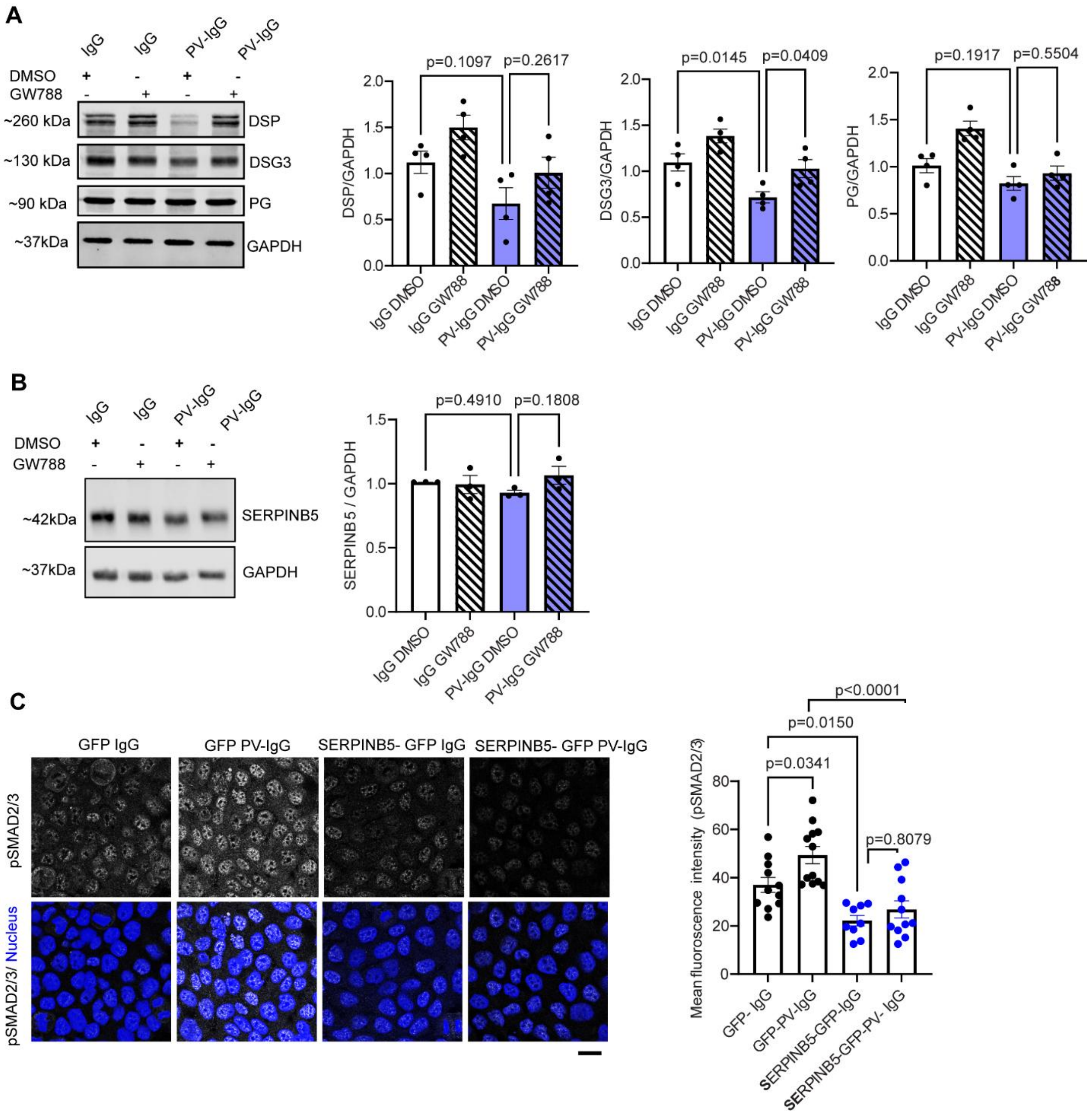

Figure S4A) Western blot analysis of HaCaT lysates from cells incubated with IgG, PV-IgG and treated with DMSO and GW788 for 24 hours, using DSP, DSG3, PG and GAPDH antibodies. Representative Western blot images and quantifications of indicated proteins (n=4) are shown. One-way-ANOVA, Sidak correction used for statistical analysis. **B)** Western blot analysis of HaCaT lysates from cells incubated with IgG, PV-IgG and treated with DMSO and GW788 for 24 hours, using SERPINB5 and GAPDH antibodies. Representative Western blot images and quantifications of SERPINB5 (n=4) is shown. One-way-ANOVA, Sidak correction used for statistical analysis. **C)** Immunofluorescence staining of HaCaT cells expressing GFP and SERPINB5-GFP, treated with IgG and PV-IgG for 24 hours, using pSMAD2/3 antibodies. Scale

bar = 10 μm. Quantification of pSMAD2/3 mean fluorescence intensity of individual cells from 3 independent experiments are shown. One-way-ANOVA, Sidak correction used for statistical analysis.

Supplementary Table 1:

Sample	Localization	Category	Gender (m/f)	Age (y)	anti-DSG3	anti-DSG1
1	skin	Ctrl	f	84	na	na
2	skin	Ctrl	m	71	na	na
3	skin	Ctrl	f	48	na	na
4	skin	Ctrl	f	97	na	na
5	skin	Ctrl	f	48	na	na
6	skin	Ctrl	f	66	na	na
7	skin	Ctrl	m	61	na	na
8	skin	Ctrl	m	74	na	na
9	skin	Ctrl	f	86	na	na
1	skin	Pemphigus vulgaris	m	69	949	143
2	skin	Pemphigus vulgaris	f	55	93	10
3	skin	Pemphigus vulgaris	f	83	141	861
4	skin	Pemphigus vulgaris	f	65	935	737
5	skin	Pemphigus vulgaris	f	65	178	3
6	skin	Pemphigus vulgaris	m	86	118	<20
7	skin	Pemphigus vulgaris	m	86	118	<20

Table 1: Table showing the patient information from control and PV. The anti-DSG3 and anti-DSG1 values indicated are titre values from ELISA assay (U/ml). Na = not detected. m/f indicates male/female respectively.
