# Supplementary figures and images for: SERPINB5/TGF-β signaling modulates desmoplakin membrane localization and ameliorates pemphigus vulgaris skin blistering
Source: JCI Insight. 2025 Oct 2;10(22):e183024. doi: 10.1172/jci.insight.183024 (PMC12643518; doi:10.1172/jci.insight.183024)

# 3A

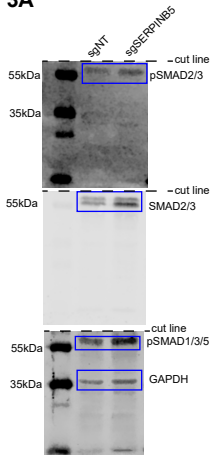

# 3D

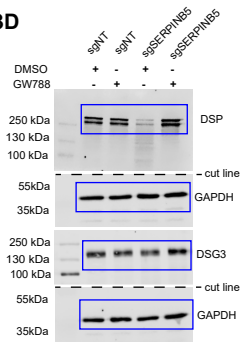

**S1A**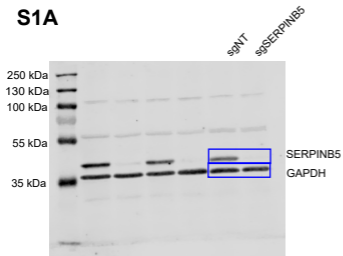**S1D**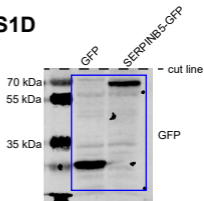

# S2A

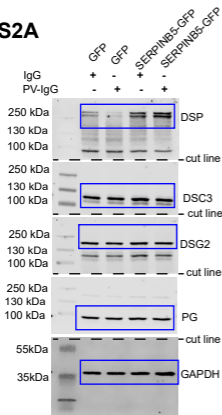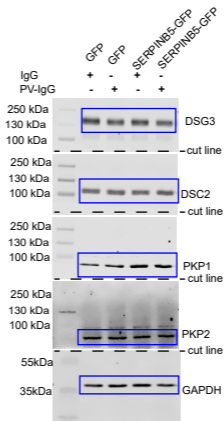

**S3A**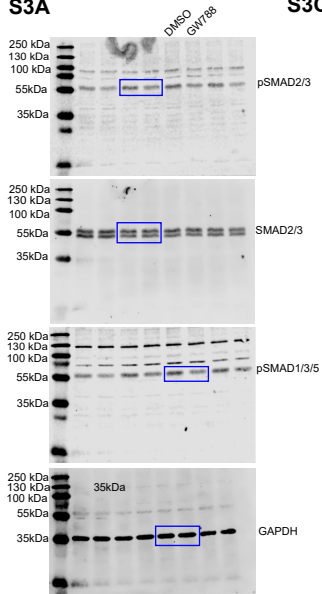**S3C**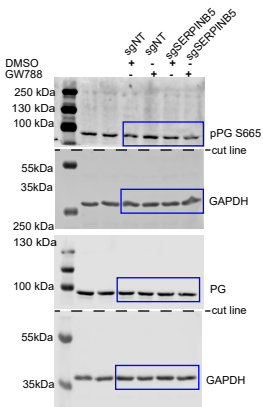**S3D**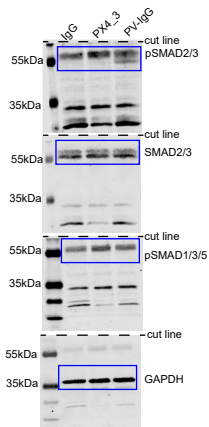**S3G**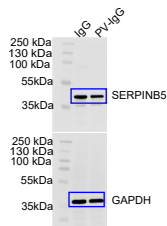

**S4A**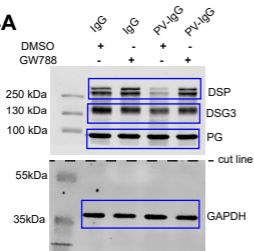**S4B**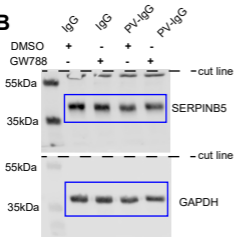

Supplement: Unedited blot and gel images [file jciinsight-10-183024-s007.pdf]
